# A Community-Driven Implementation of the Body and Soul Program in Churches in the Twin Cities, Minnesota, 2011–2014

**DOI:** 10.5888/pcd14.160386

**Published:** 2017-03-23

**Authors:** Erika Ashley Pinsker, Andrew W. Enzler, Megan C. Hoffman, Kathleen Thiede Call, Sylvia Amos, Alfred Babington-Johnson, Kolawole Stephen Okuyemi

**Affiliations:** 1 University of Minnesota Center for Health Equity, Minneapolis, Minnesota; 2 Minneapolis VA Health Care System Center for Chronic Disease Outcomes Research; 3 Stairstep Foundation, Minneapolis, Minnesota; 4 University of Minnesota Program in Health Disparities Research, Minneapolis, Minnesota

## Abstract

**Background:**

African Americans have high disease and death rates due to cancer and cardiovascular disease. Health promotion efforts to improve diet have the potential to reduce these rates.

**Community Context:**

Given their importance in the community and the extent of their reach, churches are effective avenues for health promotion efforts targeting African Americans. The objectives of this project were to promote healthy eating among African American church members, engage African American churches in the implementation of Body and Soul (an evidenced-based program that encourages healthy eating), and implement the program in the community with minimal resources.

**Methods:**

From 2011 through 2014 we conducted a community engagement project to implement the 12-week Body and Soul program, which includes demonstrations of healthy recipes and peer counseling, in 20 churches. Participants (n = 310) completed baseline and follow-up surveys on their eating habits and experience with peer counseling. Church coordinators (n = 11) completed a survey evaluating the program.

**Outcome:**

Participants’ weekly servings of fruit (baseline, 4.3; follow-up, 5.4; *P* < .001) and vegetables (baseline, 4.5; follow-up, 5.3; *P* < .001) increased. Church coordinators reported enthusiasm about Body and Soul at their church, and 10 of 11 church coordinators indicated that their pastor encouraged members to attend Body and Soul events. Program success was promoted by engaging the pastor in program activities and by scheduling events soon after church services. Implementation challenges were variation in peer counseling among churches and low turnout at follow-up events.

**Interpretation:**

The project was successfully implemented in the 20 churches, and increases in healthy eating were observed. This project demonstrated that Body and Soul can be implemented in communities with little funds or other resources.

## Background

Despite declining rates of cancer deaths among adults in the United States, rates for African American men are 27% higher than for white men and rates for African American women are 14% higher than for white women (1). Disparities also exist in the rates of cardiovascular health between African Americans and whites. Death rates from coronary heart disease are 20% higher among African American men and 19% higher among African American women. For stroke, death rates are 30% higher for African American men and 22% higher for African American women (2). Health promotion efforts that target risk factors for these diseases are needed to reduce the disparities in mortality.

Unhealthy diet is a risk factor for both cancer and cardiovascular disease (1,3). African Americans’ diets tend to be of poorer quality than diets of other populations in the United States, and African Americans tend to consume less fruit, vegetables, milk, and whole grains (4,5). Health promotion efforts that target healthy eating among African Americans promote positive changes in dietary choices (6). These efforts have the potential to reduce cancer and cardiovascular disease and deaths among African Americans. A strategy for promoting healthy eating among African Americans is for researchers and communities to collaborate on the implementation of evidence-based programs (7). Community–academic partnerships are effective in implementing positive behavior change in a community by introducing culturally relevant programs that target under-resourced populations such as African Americans (8,9).

## Community Context

Churches are well-established institutions in African American communities and are important resources for spiritual guidance and for social–emotional and tangible support (10,11). Churches are a way to reach many members of the African American community and are effective avenues for health promotion interventions (10,11). There are over 320,000 African Americans and 200 churches with predominately African American congregations in Minnesota (12). Fifty-three percent of African Americans attend religious services at least once a week (13).

The first objective of this project was to promote healthy eating among African American church members. The second objective was to engage African American churches in implementing Body and Soul, a program designed to promote healthy eating among their adult church members, which targets church policies, leadership, coordinators, and congregants. We also sought to determine whether the program could be successfully implemented in the community with minimal resources (eg, by using existing church staff and little funds). To measure the outcome of engagement efforts, we conducted an evaluation following completion of the project of each level of the Body and Soul program. 

## Methods

### Program

The evidence-based Body and Soul program was developed by the National Cancer Institute as part of its work with African American churches to increase the intake of fruits and vegetables among church members (11,14). The program has been successful when implemented in large studies with external funding and support for carrying out the intervention (11,14–16).However, the program has yet to be evaluated at the community level.

### Community engagement

This study was a partnership between the University of Minnesota’s Center for Health Equity (CHE) and the Stairstep Foundation (Stairstep), which works with over 45 African American churches in the Twin Cities (the Minneapolis–St. Paul metropolitan area of Minnesota) on social and health issues. CHE leaders contacted Stairstep about partnering with them on a community health project that would be supported by the CHE grant. Stairstep agreed and invited 20 churches to participate in the project. Church leaders saw high death rates from cancer and cardiovascular disease as a pressing issue facing the community and selected the Body and Soul program. They then provided insights on how to adapt the program to their needs and implement the program in a culturally competent way.

CHE worked with Stairstep to adapt and implement the program and to resolve measurement issues. Monthly meetings between CHE, Stairstep, and church coordinators were held to discuss program progress, challenges, and findings from each church. After completion of the project, all the churches were invited to an event in the community where project findings were shared with church members, coordinators, and pastors. CHE and Stairstep also held events for church coordinators to share their thoughts on the successes and challenges of implementing the program in their churches.

### Project design

**Figure 1:**
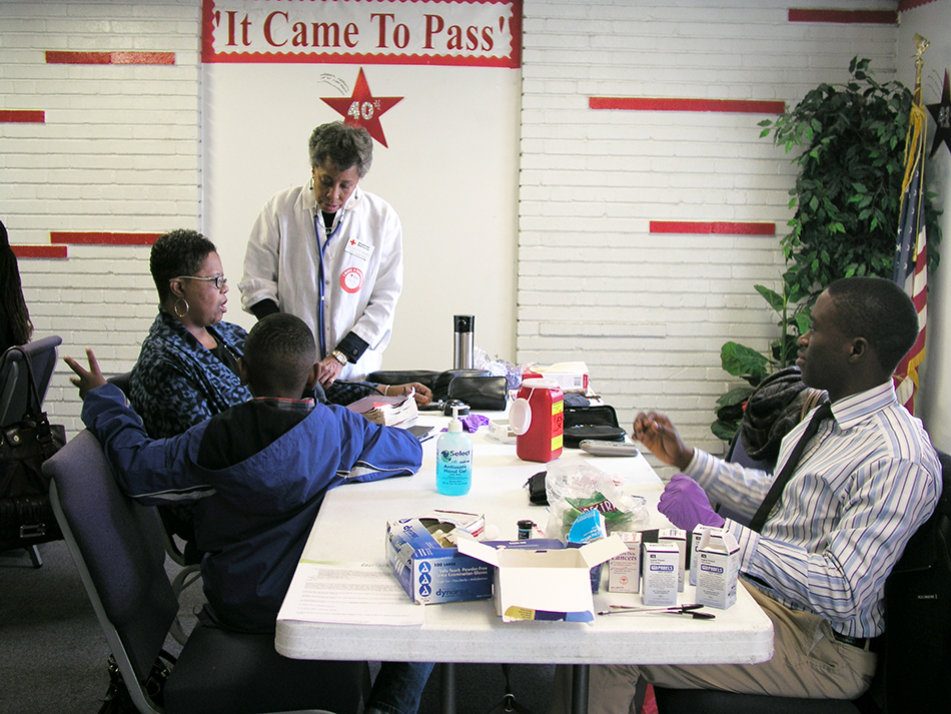
A volunteer nurse provides health information to a Body and Soul Program participant at a church kick-off event.

From 2011 through 2014, 20 churches conducted the 12-week Body and Soul program, which included kick-off and follow-up events and peer counseling sessions. The events, which occurred after Saturday or Sunday services, consisted of a study staff member demonstrating healthy recipe preparations, presenting healthy food options, and providing encouragement on healthy eating habits. Volunteer nurses provided participants with health information (eg, checking blood pressure) (Figure 1). Church coordinators at each church provided peer counseling sessions using motivational interviewing. Each church coordinator determined how to deliver the peer counseling sessions (eg, in person or by telephone, to individuals, to groups). Church coordinators were trained in motivational interviewing and provided with Body and Soul resources, including a video presentation, guide, handbook, healthy recipes, and posters. The goal was to provide at least one peer counseling session per week during the 12-week program. Church coordinators recruited participants through announcements at church programs and by word of mouth. They also posted and distributed Body and Soul posters and healthy recipes. Each church coordinator aimed to engage a minimum of 10 to 15 participants in the program. Pastors from each church were encouraged to participate in the program to lead by example and embolden others to participate. CHE provided funds for the food demonstration and food for church members at the kick-off events in addition to $24,000 per year, which was distributed among the churches to assist with costs associated with the program and/or a stipend for the church coordinators. 

**Figure 2:**
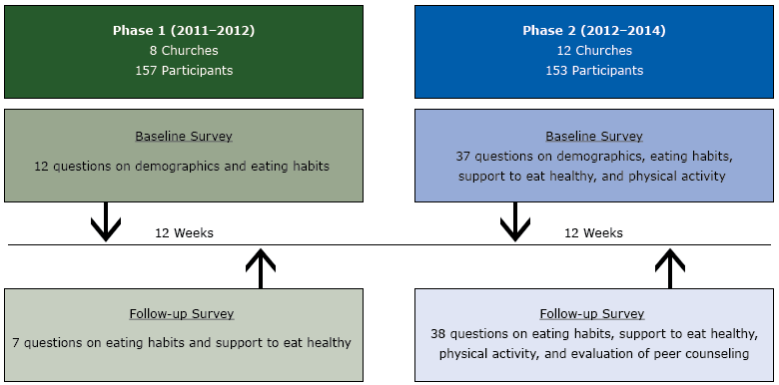
Project Design for the Body and Soul Program in the Twin Cities, Minnesota, from 2011–2014. The project was conducted in 2 phases. Phase 2 was initiated midway during the project after additional questions (based on consultant’s recommendation) were added to the surveys. There were 12 weeks between baseline and follow-up surveys in both phase 1 and phase 2.

Participants completed surveys at kick-off events and 12 weeks later at follow-up events. The surveys were adapted from Body and Soul surveys (11,14,16,17), and tailored by Stairstep leaders and church coordinators. At the request of church leaders, discussions were held with an outside consultant half-way through the project, and additional measures were added to the surveys. Churches that participated before the surveys were modified were considered phase 1 participants. Churches that participated after the surveys were modified were considered phase 2 participants (Figure 2). 

Eleven church coordinators who attended a follow-up meeting with CHE and Stairstep at the end of the project completed an 18-item survey evaluating the Body and Soul program. Six of the church coordinators were from phase 1 and 5 were from phase 2. Church members in phase 2 completed an evaluation of peer counseling as part of the follow-up survey.

### Measures

Participants were asked to report their sex, age, marital status, and highest education level completed. They were asked how many times in the past month they ate at a fast food restaurant and how many servings they consumed of a certain food, per week (0, 1–2, 3–4, 5–6, 7–8, or ≥9). Foods included were sweetened beverages (8 oz or 1 cup), 100% fruit juice (8 oz or 1 cup), fruit (1/2 cup), green salad (2 cups), and vegetables (1/2 cup, excluding white potatoes) (14,18). These variables were treated as continuous (eg, a participant who reported 1–2 servings was assigned a value of 1.5).

We used 10 items to measure self-efficacy to eat more fruits and vegetables (eg, confidence to prepare good-tasting meals that contain fruits and vegetables) (15,17). Answer options ranged from 1 (not at all confident) to 4 (very confident). The items were summed and the resulting scale ranged from 10 to 40. The Cronbach’s α for these 10 items at baseline and follow-up was 0.94, indicating excellent reliability.

Low-fat and high-fat vegetable preparation practices were each measured using 4 items that assessed how often participants carried out certain vegetable preparation practices (15,19,20). Low-fat practices included steaming or microwaving and broiling or grilling. High-fat practices included deep frying in oil and adding butter, margarine, or oil. Answer options ranged from 1 (never) to 4 (always). The items in each scale were summed and the resulting scales ranged from 4 to 16. The Cronbach’s α for the low-fat items was 0.52 at baseline and 0.59 at follow-up, indicating poor reliability. The Cronbach’s α for the high-fat items was 0.58 at baseline, indicating poor reliability, and 0.68 at follow-up, indicating acceptable reliability.

Participants were asked how much encouragement they got from family, friends or work colleagues, and church members to eat more fruits and vegetables, ranging from 1 (none) to 4 (a lot). Participants were also asked how many times a month fruits and vegetables were served at church functions (never, 1–2, 3–5, and >5), which was treated as a continuous variable (eg, a participant who indicated 1 to 2 times a month was assigned a value of 1.5).

Participants were asked how active (eg, hobbies, work, social activity) and physically active (eg, brisk walking, swimming, recreational sports) they were during the previous 2 weeks, ranging from 1 (not at all active) to 6 (extremely active). Participants were also asked how many minutes a day they walked for exercise. Extreme outliers for minutes of walking exercise were replaced by the next highest value (7 outliers).

### Program evaluation 

Participants were asked to report the number of peer counseling sessions they attended and how they felt about a series of statements (eg, “the peer counselor listened to me.”) that probed their response to conversations with a peer counselor. Answer options ranged from 1 (not true at all) to 4 (very true).

Church coordinators were asked to rate how much they agreed or disagreed with a series of statements (eg, “the pastor was involved in Body and Soul at my church.”). Answer options ranged from 1 (strongly disagree) to 5 (strongly agree). They were also asked to indicate the ways in which their pastor was involved in the program (eg, the pastor was present at Body and Soul events), whether the Body and Soul program at their church included peer counseling sessions (in person or by telephone, in a group or individually), if the amount of fruit and vegetables served at church events had increased, and if the amount of fried food, sweets, and snacks served had decreased. Last, they were asked how many sessions took place during the 12-week program (none, 1–2, 3–4, or ≥5)

### Analyses

Descriptive statistics were performed for the demographics, which were examined by follow-up status (completed baseline and follow-up vs. completed baseline only). We performed χ^2^ tests to examine each demographic variable by follow-up status. Descriptive statistics were performed for eating habits, support to eat healthfully, and physical activity at baseline and follow-up. Paired *t* tests were conducted to examine changes from baseline to follow-up. Participants had to complete both the baseline and follow-up surveys to be included in the paired *t* tests. Descriptive statistics were examined for the evaluation of peer counseling by church members and evaluation of the program by church coordinators.

## Outcome

Twenty churches and 310 church members participated in the program; 305 participants (98%) completed the baseline survey, 194 (63%) completed the follow-up survey, and 189 (61%) completed both the baseline and follow-up surveys. Of those 189, most were female (77%) and most were aged from 45 to 64 years (59%) (Table 1). Church members who completed the baseline survey but not the follow-up survey were slightly younger than those who completed both, but we found no other differences between the 2 groups.

**Table 1:** Differences in Baseline Demographic Characteristics Among Church Members (N=310) at 20 Churches by Follow-up Status, Twin Cities, Minnesota, 2011–2014^a^

Variable	Completed Baseline and Follow-Up Surveys	Completed Baseline Survey Only	*P* Value^b^
**Sex^a^**	189	109	.83
Male	43 (23)	26 (24)	
Female	146 (77)	83 (76)	
**Age^a^, y**	180	107	.01
≤24	9 (5)	16 (15)	
25–44	32 (18)	26 (24)	
45–64	106 (59)	53 (50)	
≥65	32 (18)	12 (11)	
**Marital status^a^**	182	111	.23
Married or living with a partner	84 (46)	41 (37)	
Divorced or separated	40 (22)	24 (22)	
Single or widowed	58 (32)	46 (41)	
**Education^a^**	177	107	.89
High school/GED, or less	57 (32)	32 (30)	
Some college or technical school	57 (32)	38 (35)	
College graduate	63 (36)	37 (35)	

Abbreviation: GED, general education development.

a Values are n or n (%). Totals are number of participants who answered this survey question. Numbers do not total 310 because not all participants answered each question.

b χ^2^ tests used to calculate the *P* values.

The average weekly servings of fruit (baseline, 4.3; follow-up, 5.4; *P* < .001), green salad (baseline, 3.0; follow-up, 3.7; *P* < .001), and vegetables (baseline, 4.5; follow-up, 5.3; *P* < .001) increased from baseline to follow-up (Table 2). Self-efficacy to eat more fruits and vegetables (baseline, 27.6; follow-up, 30.2; *P* = .01 [scale 10–40]), support to eat healthfully from family members (baseline, 2.5; follow up, 2.8; *P* = .04 [scale 1–4]), and level of physical activity in the previous 2 weeks (baseline, 2.9; follow up, 3.4; *P* = .01 [scale 1–6]) also increased from baseline to follow-up.

**Table 2:** Characteristics of Church Members (N = 189) from Baseline to Follow-up at 20 Churches, Twin Cities, Minnesota, 2011–2014^a^

Variable	No. of Respondents	Baseline	Follow-up	*P* Value^b^
**Eating habits**				
Number of meals at fast food restaurant, past month^c^	94	3.2 (2.5)	2.8 (2.4)	.21
Servings of sweetened beverages, per week	77	3.3 (2.9)	2.6 (2.6)	.06
Servings of 100% fruit juice, per week^d^	168	3.0 (2.5)	3.4 (2.8)	.03
Servings of fruit, per week^d^	166	4.3 (2.6)	5.4 (2.6)	<.001
Servings of green salad, per week^d^	166	3.0 (2.1)	3.7 (2.5)	<.001
Servings of vegetables, per week^d^	168	4.5 (2.6)	5.3 (2.5)	<.001
Self-efficacy to eat more fruits and vegetables, scale 10–40^e^	70	27.6 (8.1)	30.2 (7.3)	.01
Low-fat vegetable preparation practices, scale 4–16^f^	69	8.3 (2.0)	9.0 (2.3)	.02
High-fat vegetable preparation practices, scale 4–16^f^	74	8.3 (1.8)	7.9 (2.1)	.13
**Support to eat healthfully**				
Level of encouragement to eat fruits and vegetables				
From family, scale 1–4^g^	77	2.5 (1.2)	2.8 (1.1)	.04
From friends or work colleagues, scale 1–4^g^	78	2.4 (1.2)	2.6 (1.0)	.19
From church members, scale 1–4^g^	79	3.0 (1.1)	3.2 (0.9)	.16
Number of times fruits and vegetables are served at church, per month	68	2.3 (1.6)	3.0 (1.7)	<.001
**Physical activity**				
Level of activity, past 2 weeks, scale 1–6^h^	80	3.5 (1.4)	3.6 (1.3)	.38
Level of physical activity, past 2 weeks, scale 1–6^h^	78	2.9 (1.3)	3.4 (1.4)	.01
Walking exercise, minutes per day	59	37.6 (53.9)	46.5 (58.1)	.17

a Values are mean (standard deviation) unless otherwise indicated. Included participants completed both baseline and follow-up surveys. All variables were measured at baseline and follow-up of phase 2. Some variables were also measured in phase 1, as indicated.

b Paired *t* tests were conducted to calculate *P* values.

c Measured at baseline and follow-up during phase 2. One church in phase 1 was also asked this question.

d Measured at baseline and follow-up during phase 1 and phase 2

e Self-efficacy to eat more fruits and vegetables was measured using 10 items (eg, confidence to prepare good-tasting recipes that contain fruits and vegetables). Answer options ranged from 1 = not at all confident to 4 = very confident. The items were summed and the resulting scale ranged from 10 to 40.

f Low-fat and high-fat vegetable preparation practices were each measured using 4 items that assessed how often participants carried out certain vegetable preparation practices. Low-fat practices included steaming or microwaving and broiling or grilling. High-fat practices included deep frying in oil and adding butter, margarine, or oil. Answer options ranged from 1 (never) to 4 (always). The items in each scale were summed and the resulting scales ranged from 4 to 16.

g Participants were asked how much encouragement they got from family, friends or work colleagues, and church members to eat more fruits and vegetables, ranging from 1= none to 4 = a lot.

h Participants were asked how active (eg, hobbies, work, or social activity) and physically active (eg, brisk walking, swimming, recreational sports) they have been during the past 2 weeks, ranging from 1 = not at all active to 6 = extremely active.

The average number of peer counseling sessions attended by participants was 4.6 (Table 3). On a scale of 1 (not true at all) to 4 (very true) participants said that the peer counselors helped them to think differently about their health habits (mean = 3.6), listened to them (mean = 3.7), and were supportive and encouraging (mean = 3.8). On a scale of 1 (strongly disagree) to 5 (strongly agree) church coordinators felt prepared to do peer counseling with participants (mean = 4.4) and indicated that there was a great deal of enthusiasm about Body and Soul at their church (mean = 4.2).

**Table 3:** Evaluation of the Body and Soul Program by Church Members (N = 92) and Coordinators (N = 11) at 20 Churches, Twin Cities, Minnesota, 2011–2014^a^

Variable	No. of Respondents	Mean (Standard Deviation)
**Evaluation of Peer Counseling by Church Members**		
**Number of peer counseling sessions attended**	66	4.6 (4.3)
**On a scale from 1 (not true at all) to 4 (very true)**		
Conversations with the peer counselor helped me think differently about my health habits	67	3.6 (0.6)
Peer counselor understood what I was saying	68	3.6 (0.7)
Peer counselor listened to me	66	3.7 (0.6)
Peer counselor rushed me through conversations	68	1.5 (1.0)
Peer counselor asked too many questions	65	1.7 (1.1)
Peer counselor asked permission before giving me information or advice	59	3.4 (1.0)
Peer counselor was supportive/encouraging	65	3.8 (0.5)
**Evaluation of Body and Soul by Church Coordinators**		
**On a scale from 1 (strongly disagree) to 5 (strongly agree) **		
It was easy to recruit participants to the Body and Soul kick-off event	11	3.9 (1.1)
It was easy to get participants to attend the follow-up event	11	3.7 (1.0)
It was easy to get enough volunteers to run the Body and Soul events	11	3.9 (1.1)
I felt well prepared to do peer counseling with Body and Soul participants	11	4.4 (0.8)
There was a great deal of enthusiasm about Body and Soul at my church	11	4.2 (0.6)
The pastor was involved in Body and Soul at my church	11	4.5 (0.7)

a Church members who evaluated peer counseling were from phase 2. Church coordinators who evaluated the Body and Soul Program were from 6 phase 1 churches and 5 phase 2 churches. Not all participants answered each question.

Most church coordinators (10 of 11) indicated their pastor encouraged members to attend the Body and Soul events; 7 of 11 said their pastor was present at Body and Soul events; all 11 said the amount of fruits served at church events increased; and 9 of 10 said the amount of vegetables had increased (Table 4). Of the 11 church coordinators, 2 initiated 0 peer counseling sessions, 3 initiated 1 to 2 peer counseling sessions, 2 initiated 3 to 4 peer counseling sessions and 4 initiated 5 or more peer counseling sessions during the 12 weeks.

**Table 4:** Reported Outcomes of the Body and Soul Program by Church Coordinators (N = 11) at 11 Churches, Twin Cities, Minnesota, 2011–2014^a^

Outcome	No. of Respondents	No. of Respondents Endorsing Outcome
**Pastor’s participation**		
Spoke about Body and Soul events during service	11	6
Encouraged members to attend Body and Soul events	11	10
Was present at Body and Soul events	11	7
Participated in the Body and Soul program	11	4
**Counseling**		
Any type of peer counseling	11	9
Telephone counseling	10	5
Individual counseling	10	7
Group counseling	10	4
**Changes in food offerings at church events**		
Increase in amount of fruit served	11	11
Increase in amount of vegetables served	10	9
Decrease in amount of fried food served	11	10
Decrease in amount of sweets and snacks served	11	6

a Church coordinators who evaluated the Body and Soul Program were from 6 phase 1 churches and 5 phase 2 churches. Not all participants answered each question.

A major achievement of the project was that the Body and Soul program was successfully implemented in church communities, and the project established the potential for other churches to implement the program without investing substantial resources. However, there were challenges. One was participant recruitment. Some churches had low turnout and other churches over-recruited. An additional challenge was that the dose and method of peer counseling varied from church to church. Some church coordinators made weekly calls, monthly calls, or only a few calls, while others had regular face-to-face meetings with individuals or a group. Another challenge was that many participants did not show up to the follow-up event because of travel problems, illness, and other day-to-day constraints, and therefore did not complete the follow-up survey.

Success of the program was increased by the enthusiasm of the church coordinators and the engagement of the pastors, who encouraged congregants to participate in the program and were present at Body and Soul events. Success of the program was also promoted through the timing of the events. Because the kick-off and follow-up events were held at each church on a Saturday or Sunday after weekly services, it was convenient for church members to participate in Body and Soul. The timing of the events was also helpful in encouraging the pastors to be present at the events.

One unexpected issue regarding the timing of the events was that many participants fasted before church and were quite hungry after church services, and we did not provide a full meal at the events. An additional unexpected issue was that many participants did not complete the follow-up survey: only 63% did so. However, examination of the baseline demographic characteristics revealed little differences between those who completed follow-up and those who did not (Table 1). Another unexpected issue was that we were encouraged to expand the survey after 8 of the 20 churches had already completed the program; therefore, we obtained less information on participants from the first 8 churches. An unexpected failure is that church coordinators often did not reach the goal of delivering 12 peer counseling sessions because of time constraints. Participants completed an average of only 4.6 sessions.

Despite the challenges of implementing this program, communication between CHE and Stairstep was effective. Leaders from CHE and Stairstep met in person monthly at Stairstep and kept in regular communication via email and telephone. Stairstep was the point of contact for the church coordinators and set the tone for church coordinators’ involvement in and enthusiasm about the project. Community participation varied from church to church and was driven in large part by the leadership and passion of the church coordinators. Since the end of the project, Stairstep expanded its network of church coordinators to 23 and meets with them monthly to provide information and training that allows church coordinators to customize health strategies for their churches.

## Interpretation

This project focused on promoting healthy eating among African American church members and on engaging 20 African American churches in implementing the Body and Soul program. The program had success in increasing the fruit and vegetable intake among the participants. Additionally, church coordinators were highly satisfied with the program and noted increases in the amount of fruits and vegetables served at church functions as a result of the program. Our findings are consistent with previous Body and Soul studies that found increases in fruit and vegetable consumption and changes to church policies around healthy eating (11,14–16). Although previous Body and Soul studies were well-funded interventions with professional and research staff, our project was implemented and conducted by church coordinators with little external support (14–16).

This project demonstrated that the Body and Soul program can be implemented and succeed in a low-cost, low-resource, real-world setting. It would be worthwhile for future projects to investigate the longevity of the results of the Body and Soul program in the community by following up with churches to see whether the integrity of the program was maintained or whether changes in church practices regarding serving healthier food at functions were sustained over time. It would also be worthwhile for future projects to determine which factors (eg, peer counseling, pastor involvement, fruit and vegetable options at church functions) contribute most to a successful and sustainable Body and Soul program.

Communities that are interested in implementing the Body and Soul program should aim to generate enthusiasm among church coordinators and should engage pastors in promoting and participating in the program, because they serve as an inspiration to their congregants. To avoid low turnout, congregations should ensure that kick-off and follow-up events are at a time when most congregants can attend. Last, communities should ensure that church coordinators have the time and ability to provide 12 weekly peer counseling sessions, given that participants in our study found these sessions to be helpful. If these aims are met, greater increases in fruit and vegetable consumption may be observed.

Despite the challenges, the project was a success, and the primary objectives of the project were achieved: healthful eating habits increased among congregants, and the Body and Soul program was implemented in 20 African American churches where it touched the lives of more than 300 congregants. Furthermore, if the implementation and evaluation of the program serves to convince church leaders of the benefits of the program, Body and Soul may be sustained and could continue to yield rewards.
